# Delayed Diagnosis of Calcaneal Stress Fracture: A Case Report

**DOI:** 10.7759/cureus.83161

**Published:** 2025-04-29

**Authors:** Ragul Rajivan, Jamie Hind, Suzani Shrestha, Neil Ashwood

**Affiliations:** 1 Orthopaedics, Oxford University Hospitals National Health Service (NHS) Foundation Trust, Oxford, GBR; 2 Trauma and Orthopaedics, Walsall Manor Hospital, Walsall, GBR; 3 Trauma and Orthopaedics, University Hospitals of Derby and Burton National Health Service (NHS) Foundation Trust, Derby, GBR

**Keywords:** calcaneal stress fracture, calcaneum, delayed diagnosis, diagnosis, fracture, heel pain, mri, stress fracture, x-ray

## Abstract

The calcaneus is a key component of the hindfoot, susceptible to stress fractures due to its role in bearing significant axial loads. These fractures often result from repetitive forces, yet their diagnosis can be challenging due to overlapping symptoms with more common causes of heel pain. Early recognition of calcaneal stress fractures is essential to prevent complications like non-union and the need for surgical intervention. Given that initial radiographs may appear normal, advanced imaging techniques such as magnetic resonance imaging (MRI) should be considered when clinical suspicion remains high.

Here, we report a 57-year-old woman who presented with acute left heel pain following a minor trauma. Initial X-rays showed no acute bony injury, leading to a soft tissue injury diagnosis. However, persistent pain prompted further evaluation, revealing a stress fracture of the left calcaneum upon MRI. Conservative management with non-weight-bearing and an ankle boot led to symptomatic improvement.

As calcaneal stress fractures often elude initial diagnosis due to their early radiographic normalcy, this report postulates a management pathway for patients presenting with heel pain to avoid a delayed diagnosis.

## Introduction

The calcaneus forms part of the hindfoot with the talus, the largest of the seven tarsal bones [1]. Due to its posterior and inferior position, it accommodates much of the axial load of the body and is therefore susceptible to stress injury, in addition to acute trauma [1]. Tarsal fractures account for 2% of all fractures, of which calcaneal fractures account for 60% of all fractured tarsal bones [1]. Stress fractures usually occur in the bone as a result of repetitive forces, the accumulation of which leads to macrostructural failure. Calcaneal stress fractures, though uncommon, often result from repetitive loading activities, especially in individuals with risk factors such as osteoporosis, prolonged corticosteroid use, or sudden increases in physical activity [2].

These fractures are typically a result of repetitive, suboptimal forces, which damage bone microscopically over time [2,3]. The accumulation of such microscopic damage can result in reactive bone oedema, which is primarily visible by magnetic resonance imaging (MRI) [2]. In the early stages of presentation, radiographic abnormalities may not be present, as up to 85% of X-rays can be unremarkable at this time [2]. Due to the uncommon nature of this pathology and the low sensitivity of X-rays in the initial presentation of calcaneal stress fractures, early consideration in cases presenting with a high index of clinical suspicion can help aid in early recognition, diagnosis, and management. Early detection may then reduce the risk of non-union and the subsequent requirement of surgical intervention. This case presents a middle-aged woman who had a delay in diagnosis and appropriate treatment after sustaining a direct low-energy traumatic calcaneal stress fracture. As calcaneal stress fractures can often be overlooked, this case report proposes a management pathway for patients presenting with heel pain, to prevent the delayed diagnosis of calcaneal stress fractures.

## Case presentation

A 57-year-old woman presented to the emergency department (ED) with acute left-sided heel pain after stepping off a footstep at home earlier that day. On examination, she had localised tenderness over the posteromedial aspect of the hindfoot with minor erythema and reported worsening pain on mobilisation and weight-bearing, all of which should raise the clinical suspicion for a possible stress fracture. She retained a good range of ankle movement and had no signs of vascular compromise or neurological deficits in the affected limb.

Her medical history included chronic asthma, for which she used corticosteroid inhalers and preventive bronchodilators, hypothyroidism managed with thyroxine, and a long-standing smoking habit, common risk factors associated with impaired bone mineral density and stress fractures. However, less commonly associated, this patient led a sedentary lifestyle and did not engage in regular physical activity.

Clinically, the pain was deep and aching in nature, contrasting with the sharp, stabbing pain typical of plantar fasciitis, which is often worse with the first steps in the morning and tends to improve with activity. Similarly, the patient's presentation differed from Achilles tendonitis, which is characterised by swelling and tenderness along the tendon. Her pain was localised directly over the heel bone and worsened with activity, features more consistent with a calcaneal stress fracture.

Initial plain radiographs of the foot showed no signs of acute bony injury, and the diagnosis was presumed to be a soft tissue injury. The patient was discharged with analgesia and safety-netting advice, including instructions to return if symptoms worsened.

One week later, she re-presented to the ED with persistent left heel pain, now more pronounced on weight-bearing. Examination again revealed tenderness over the medial heel with minor swelling, but neurological and vascular assessments remained unremarkable. She was referred to the Fracture Clinic for further evaluation.

**Figure 1:**
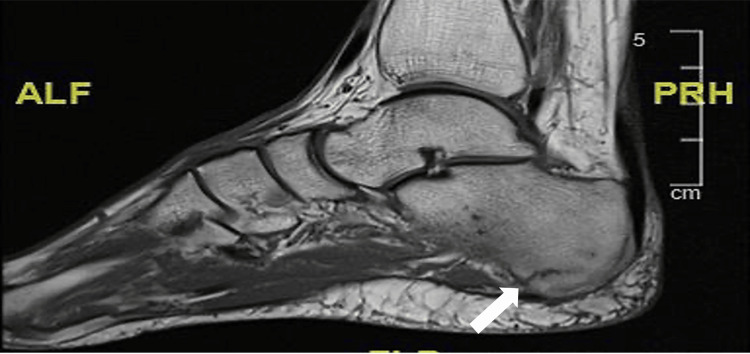
Original T1-weighted MRI of the patient's left ankle. Incomplete banding of low signal in the plantar cortex of the posterior calcaneus, consistent with bone stress injury, highlighted with white arrow. MRI: magnetic resonance imaging; ALF: anterior lateral foot; PRH: posterior hindfoot

Given the persistence of symptoms and the initial radiographs being inconclusive, an MRI was performed three weeks post-injury, which confirmed a stress fracture of the left calcaneus, underscoring the modality's sensitivity in detecting early osseous injuries (Figure 1).

She was managed conservatively with non-weight-bearing advice and the use of an orthopaedic walking boot for four to six weeks. At her six-week follow-up, she reported significant improvement in pain and swelling, indicating the successful conservative management of the fracture.

## Discussion

The most common causes of heel pain include plantar fasciitis, heel spurs, Achilles tendonitis, bursitis, heel pad syndrome, tarsal tunnel syndrome, and nerve entrapment [4]. Less commonly, heel pain may be attributed to osteomyelitis or tumours or secondary to systemic conditions [4]. Stress fractures of the calcaneus are an uncommon and rare cause of heel pain, and the diagnosis is often missed because of its similarity to the more common causes attributed to heel pain. This case helps to emphasise that stress fractures of the calcaneus should be taken into account whenever dealing with individuals presenting with heel pain.

Stress fractures typically occur due to the inability of osteocytes to adequately respond to repeated biomechanical stress, resulting in microscopic fractures. Stress fractures can be subsequently divided into fatigue reaction stress fractures, where there is excessive strain applied to structurally normal bone, or insufficiency reaction stress fractures, whereby normal stress is applied to structurally impaired bone [5].

Unlike traumatic fractures, radiographic abnormalities may not be present in the early stages of stress fractures. X-ray may lack sensitivity and thus be ineffective in diagnosing a stress fracture in its initial phase, as up to 85% can be negative at the time of symptom onset [2,6]. However, a fracture line may be revealed on repeat X-rays a few weeks later, due to callus formation around the fracture site [6]. The majority of calcaneal stress fractures are missed on initial X-rays as they typically appear normal during the initial phase [6,7]. Plain films will often show a sclerotic or radiolucent line only after 2-3 weeks of symptoms [8,9]. In contrast, MRI allows for a more accurate definition of early changes associated with osseous stress injury and is recognized as a preferred imaging modality for stress fractures [9].

Early recognition of calcaneal stress fractures is important for optimal patient-centred treatment as weight-bearing in patients with a stress fracture can complicate healing and lead to prolonged pain. Thus, it is important to appropriately investigate patients presenting with acute heel pain to avoid delayed diagnosis and treatment. The most common signs and symptoms to consider when assessing for calcaneal stress fractures in patients presenting with heel pain include generalised pain in the heel area that is worse after activity, oedema of the heel area, positive calcaneal compression test, and positive Mondor sign (haematoma formation along the sole of the foot) [1,10].

Table 1 shows a review of calcaneal stress fracture case reports regarding the history and timing of diagnosis.

**Table 1:** Calcaneal stress fracture case reports summarised by presenting complaint and mode of diagnosis. GP: general practitioner; MRI: magnetic resonance imaging; CT: computed tomography; ED: emergency department

SN	Case report	Case	Mode of diagnosis
1	Imerci et al., 2012 [11]	44y/F with bilateral heel pain. Initially treated as Achilles tendonitis and plantar fasciitis.	Delayed diagnosis. Confirmed stress fracture later with MRI.
2	Eggink et al., 2014 [12]	47y/F with a 3-week history of non-traumatic heel pain.	Delayed diagnosis. Ultrasound scan normal. Confirmed by repeat X-rays after 6 weeks post-injury.
3	Engbjerg et al., 2021 [13]	15y/M presented to GP with a 4-day history of heel pain. Diagnosed as a heel spur. Presented again 4 days later (8 days after the onset of pain) and treated as inflamed Achilles tendon.	Delayed diagnosis. Following his third presentation to the GP for persistent heel pain (12 days after symptom onset), the patient was referred to ED, and a calcaneal stress fracture diagnosis was confirmed on X-ray.
4	Erdem et al., 2017 [14]	43y/M presented with a 5-day history of left heel and knee pain following 6 weeks of training for running.	No delay in diagnosis. Diagnosed on the first visit to trauma and orthopaedic department with CT, though initial X-ray did not show calcaneal stress fracture.
5	Mattingly et al., 1960 [15]	48y/F, with a 3-week history of left ankle pain and a 3-day history of swollen ankle.	Delayed diagnosis. The initial X-ray was negative. The patient was treated as a case of suspected tuberculous arthritis. Repeat X-ray 3 weeks later showed a left calcaneal stress fracture.
6	Ficek et al., 2020 [16]	42y/F presented with a 2-month history of left ankle pain after Zumba classes.	No delay in diagnosis. Same-day MRI showed calcaneal stress fracture with bone marrow oedema and swelling of the adjacent tissue.
7	Miki et al., 2014 [17]	Review of 5 cases of calcaneal stress fractures in post-total hip and total knee arthroplasty patients.	Delayed diagnosis. Initial X-rays of two patients were normal (40%). Diagnosis was made by subsequent X-rays.
8	Moe et al., 2006 [18]	48y/F with left heel pain. Initially treated as plantar fasciitis.	Delayed diagnosis. MRI confirmed calcaneal stress fracture in later visits.
9	Kose et al., 2016 [19]	33y/F with a 2-month history of non-traumatic heel pain and a past medical history of celiac disease.	Delayed diagnosis. The initial X-ray was normal. The patient was treated as a case of plantar fasciitis. Later MRI confirmed calcaneal stress fracture.
10	Pearce et al., 2011 [20]	13y/F basketball player with non-traumatic right foot pain.	No delay in diagnosis. MRI confirmed anterior process fracture of the calcaneus.

The presenting complaint of heel pain and associated swelling, seen in this case study, is similar to the vast majority of cases summarised in Table 1. However, in a few cases, patients also presented with ankle or foot pain. Upon reviewing the 10 case reports, delayed diagnosis was noted in seven reports (70%), six of which were due to negative X-ray findings on initial imaging. This suggests that as a first-line investigation, initial X-rays may fail to provide a definitive diagnosis and additional imaging is required. Upon final diagnosis, these patients all required conservative management with an average follow-up duration of six weeks. Following conservative management, patients reported either no or significantly improved pain whilst weight-bearing or mobilising, along with a subsequent return to daily activities. These cases illustrate that timely conservative management, when initiated appropriately, can lead to symptom resolution within four to six weeks.

**Figure 2:**
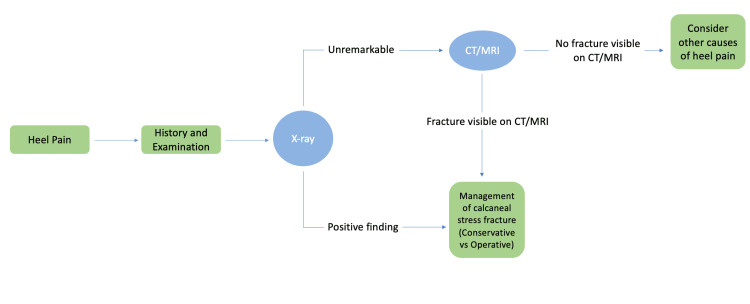
Proposed investigation and management pathway designed by authors for suspected calcaneal stress fractures. MRI: magnetic resonance imaging; CT: computed tomography

Hence, the authors propose a management pathway for heel pain, as shown in Figure 2, to avoid the delayed diagnosis of calcaneal stress fractures.

This case underscores the diagnostic challenge of calcaneal stress fractures, which may be overlooked due to normal initial radiographs and symptom overlap with more common causes of heel pain. Particular attention should be paid to older adults, smokers, and patients on corticosteroids, who may be more prone to insufficiency-type stress fractures. In this case study, the patient's corticosteroid use and hypothyroidism may have contributed to bone fragility and should have raised the suspicion of an insufficiency-type stress fracture.

Although stress fractures typically heal with conservative management, complications such as non-union or, in rare cases, heterotopic ossification may occur during the healing process. Awareness of these outcomes is essential in tailoring follow-up and rehabilitation protocols. Establishing a structured management pathway for heel pain can help reduce the risk of misdiagnosis and delayed diagnosis, ultimately improving patient outcomes.

## Conclusions

Stress fractures of the calcaneus are frequently missed on initial presentation as the presenting symptoms are similar to other more common causes of heel pain. Unfortunately, initial simple radiographs are not sufficient to appreciate these fractures in the early stages. Implementing a structured management pathway that incorporates advanced imaging modalities can facilitate the timely diagnosis and treatment of calcaneal stress fractures, potentially improving patient outcomes.
